# Gene expression analysis in NSAID-induced rat small intestinal disease model with the intervention of berberine by the liquid chip technology

**DOI:** 10.1186/s41021-021-00205-2

**Published:** 2021-07-20

**Authors:** Guanqun Chao, Qianqian Wang, Fangxu Ye, Shuo Zhang

**Affiliations:** 1 Department of General practice, Sir Run Run Shaw Hospital, Zhejiang University, Hangzhou, China; 2 Department of Gastroenterology, The First Affiliated Hospital, Zhejiang Chinese Medical University, Youdian Road No. 54, Hangzhou, 310006 China

**Keywords:** NSAID-induced small intestinal disease, Berberine, Gene, The liquid chip technology

## Abstract

**Objective:**

Investigate the effect and mechanism of berberine on the small intestinal mucosa of non-steroidal anti-inflammatory drugs (NSAIDs) related small intestinal injury.

**Materials and methods:**

Twenty-four SD rats were randomly divided into control group, model group and intervention group. The model group and intervention group were treated with diclofenac (7.5 mg/kg·d, 2/d), a total of 4 days tube feeding, and the intervention group was treated with 50 mg/kg·d intragastric administration of berberine after 2 days. The control group was treated with 7.5 mg/kg·d, 2/d 0.9% saline tube feeding. Then we screened differential expression of colonic mucosal gene by the liquid chip technology.

**Results:**

Compared with the control group, macroscopic and histology score of the model group increased significantly (*P* < 0.05), HTR4, HTR1a, F2RL3, CALCA, NPY, CRHR2, IL1b, P2RX3, TPH1, HMOX1, TRPV1, VIP, F2RL1, SLC6A4, TFF2, AQP8 content were significantly increased (*P* < 0.05), NOS1 content decreased significantly (*P* < 0.05); Compared with the model group, macroscopic and histology score of the intervention group improved significantly (*P* < 0.05), and HTR4, F2RL3, NPY, CRHR2, IL1b, VIP, AQP8 content were significantly lower (*P* < 0.05), NOS1 content increased significantly (*P* < 0.05).

**Conclusion:**

Berberine has a protective effect on NSAID-associated small intestinal injury, the mechanism may be that berberine decreases the expression of intestinal mucosa HTR4, F2RL3, NPY, CRHR2, IL1b, VIP, AQP8, and increases the expression of NOS1, that to reduce intestinal permeability and protect intestinal mucosal barrier.

## Introduction

Non-steroidal anti-inflammatory drugs (NSAIDs) is a kind of anti-inflammatory drug that does not contain steroid structure, which is widely known that have effect on anti-inflammatory, anti-rheumatism, alleviating pain, pyretolysis and anticoagulated blood and so on. With the wild application of NSAIDs in rheumatoid arthritis, cardiovascular and cerebrovascular diseases, its adverse reactions are also exposed. These side effects involve the digestive system, blood system, skin connective tissue and respiratory system, among which the most common is the adverse reactions of the gastrointestinal tract [1]. In current, NSAIDs is believed to help many patients, but only estimated 25% of them receive adequate pain relief [2] in fact. NSAID-induced enteropathy is common. It is reported that the incidence of intestinal damage is up to two-thirds [3]. NSAID-induced small intestinal disease can be manifested as mucosal congestion, edema, erosion and a superficial ulcer formation, severe cases can cause a large area of ulcers, and the clinical manifestations show as abdominal pain, abdominal discomfort, indigestion, diarrhea, gastrointestinal bleeding, perforation and even life-threatening [4].

It is reported that various damage features of the small intestine are common among long-term NSAIDs users: inflammation accounts for 60–70%, ulceration accounts for 30–40%, increased permeability is up to 70%, bleeding/anemia is about 30%, and malabsorption occupies 40–70% [5] so severe and complex the consequence is,but the mechanism of NSAID-induced small intestinal disease is still unclear. At present, the understanding of the mechanism of NSAID-associated small intestinal injury is mainly focused on the changes of cell membrane permeability, inflammatory reaction, microbial imbalance, and the change of brain gut axi [6].

A lot of research on the mechanism of NSAID-induced small intestinal injury and therapeutic method had been made through experiments and clinical capsule studies. And selective COX-2 inhibitors, prostaglandin derivatives, cytoprotective drugs, Phosphatidylcholine-NSAID, and probiotics all shown to have potential protective effects on NSAID-induced small intestinal injury. However, no new promising and standardized drugs had been developed for NSAID-associated small intestinal injury until recently [7]. Berberine, an isoquinoline alkaloid, is a major bioactive component of Coptidis Rhizome which is commonly used as an herbal medicine for treating diarrhea and bacterial and parasitic infections [8]. It has wide biological and pharmacological actions, including preventing 2,4,6-trinitrobenzene sulfonic acid (TNBS)-induced colitis [9], reducing cholesterol levels [10], improving glucose metabolism [11], attenuating autoimmune encephalomyelitis [12]. Clinically, berberine can alleviate the abdominal pain, diarrhea and other symptoms caused by NSAID-induced small intestinal injury, but the specific mechanism is unknown.

Therefore, our present study aims to screen differential expression of small intestinal mucosa gene by the liquid chip technology so as to build a gene regulatory network for NSAID- induced small intestinal disease. Meanwhile, investing the specific mechanism of berberine to prove it has effective therapeutic effects from the gene level.

## Methods

### Experimental animal

Twenty-four female Sprague Dawley (SD) rats (200–220 g,12 weeks old), provided by Animal Experiment Center of Zhejiang Chinese Medicine University, without feeding drugs or used as experimental animals, were kept in room cages at 22–24 °C, humidity< 60%, noise <50db. Rats were fed with standard diet and distilled water. The rats were acclimatized for a week before the start of the experiment, and were divided randomly into three groups, the control group (*n* = 8), the model group (*n* = 8) and the intervention group (*n* = 8). This study was reviewed and approved by the Medical ethics committee of Zhejiang Chinese Medical University. All sections of this report follow the ARRIVE Guidelines for reporting animal research.

### Groups dividing and disposal

#### Control group

8 SD normal rats in the control group was tube feeding with 7.5 mg/kg·d, 2/d 0.9% saline for 4d

#### Model group

Dissolving diclofenac sodium (Daifen, Fujisawa German companies, the National Yao Zhtmzi J20050064) into 0.9% saline. Referring to the long-term of human oral dose (150 mg/d), and converting into rats oral dose 7.5 mg/kg·d, 2/d, a total of 4 d tube feeding preparation model of intestinal injury in 8 SD rats.

#### Intervention group

After 2d of diclofenac sodium tube feeding, the berberine (Hangzhou Minsheng Pharmaceutical Group Co. Ltd) crushed dissolved in 0.9% saline, 50 mg/kg·d tube feeding to 8 SD rats for 2d.

All rats were fasted for 24 h, and put to death by intraperitoneal injection of 1%pentobarbital sodium on 5th day. Opening the rats’ abdominal cavity cutting 2 cm bowel from the ileocecal valve 5 cm, taking intestinal mucosa macroscopic and histology evaluation.

### 1.3 Macroscopic and histology evaluation

Anatomical lesion score from Wallace et al. [13] was used to calculate the damage of rats’ small intestinal mucosal: ulceration: 0 score (normal appearance); 1 score (focal hyperaemia without ulcers); 2 scores (ulceration without hyperaemia or bowel wall thickening); 3 scores (one site of ulceration with inflammation); 4 scores (at least two sites of ulceration and inflammation); 5 scores (major sites of damage extending > 1 cm along the length of the intestine). Damage extended to > 2 cm along the length of the intestine increases the score by one for each additional cm of damage. Adhesions: 0 score (no adhesions to surrounding tissue); 1 score (minor adhesions (intestine can be separated from other tissueseasily)); 2 scores (major adhesions).

Each intestinal segment was processed into paraffin. Preparing Serial paraffin sections (4 μm) and staining with hematoxylin–eosin and PAS for morphological examination. Chiu scale [14] was used to assess histologic damage by two observers. 0, normal mucosa; 1, development of sub-epithelial (Gruenhagen’s) spaces; 2, extension of the sub-epithelial space with moderate epithelial lifting from the lamina propria; 3, extensive epithelial lifting with occasional denuded villi tips; 4, denuded villi with exposed lamina propria and dilated capillaries; and 5, disintegration of the lamina propria, appearance of hemorrhageand ulceration.

### Experimental procedure

The main procedure was as below: High-throughput sequencing was conducted on independent samples. Total ribonucleic acid (RNA) was extracted from duodenal tissues through TRIzol (Invitrogen, Gaithersburg, MD, USA) one-step method. Then we did primer test and sample assay. After purification, RNA concentration was analyzed by Nanodrop (Nanodrop Technologies, Wilmington, DE) and quality testing was conducted by using BioAnalyzer (Agilent Technologies, Palo Alto, CA). SuperScript II reverse transcriptase was used to synthesize complementary DNA(cDNA). Luminex 100TM was used to detect the gene expression of intestinal mucosa.

### Statistical analysis

The data of normal distribution was demonstrated as ^−^x ± SD, and was analysed by using one-factor analysis of variance. The data of nonnormal distribution was demonstrated by median and full range with using rank sum test, *P* < 0.05 showed that the difference had statistical significance.

## Results

### Macroscopically visible lesions

**Fig. 1:**
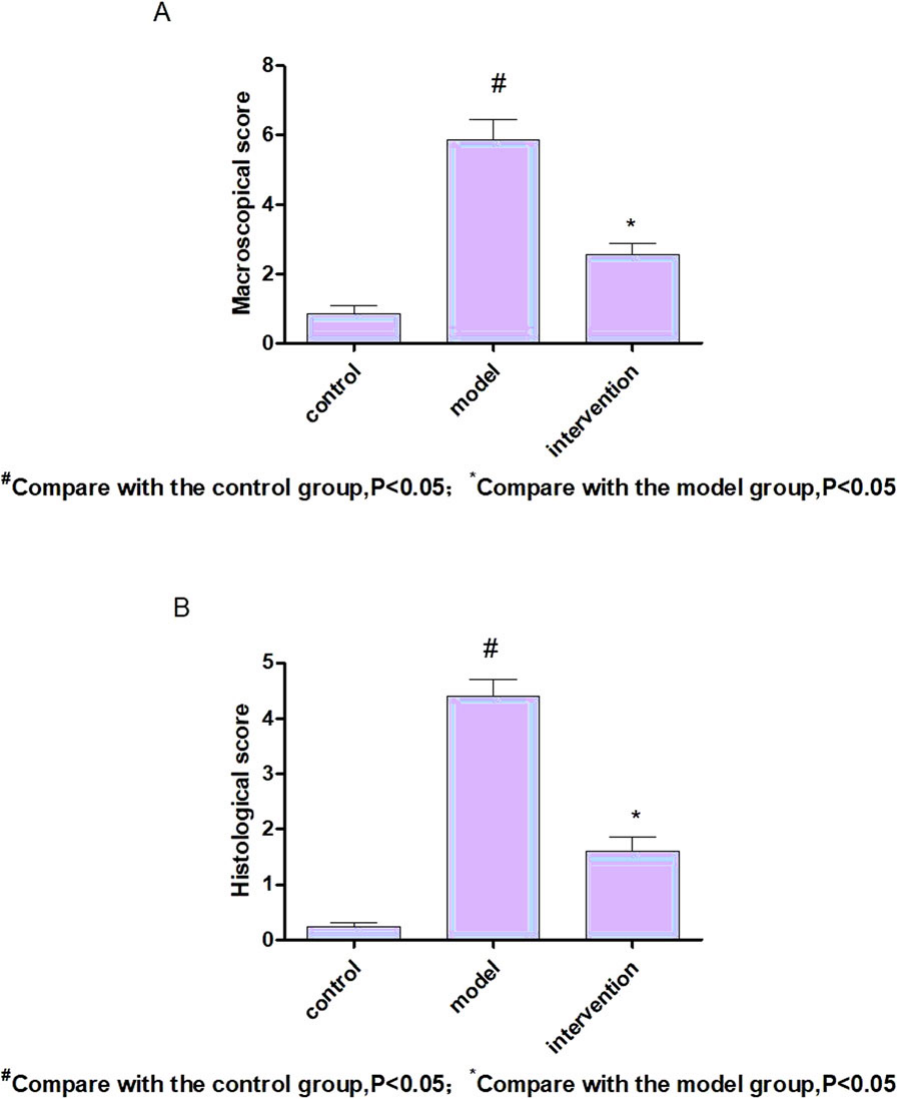
There was significant change in macroscopical scores (**A**) and histological scores (**B**), Model group rats’ injury score was higher than that of the control group (*P* < 0.05), and the intervention group was significantly lower than that of model group (*P* < 0.05)

In model group, diclofenac lead to damage including erosion and ulcer in the small intestinal mucosa. Ulcers were more frequently in ileum and predominantly located along the mesenteric border, and the berberine could improve the injury of small intestine. The model group (5.86 ± 0.59) had higher scores than the control group (0.86 ± 0.24)(*P* < 0.05). The lesion scores of small intestinal mucosal of berberine intervention group (2.57 ± 0.33) was significantly lower (*P* < 0.05) compared with the model group (Fig. 1A).

### Histologic damage

**Fig. 2:**
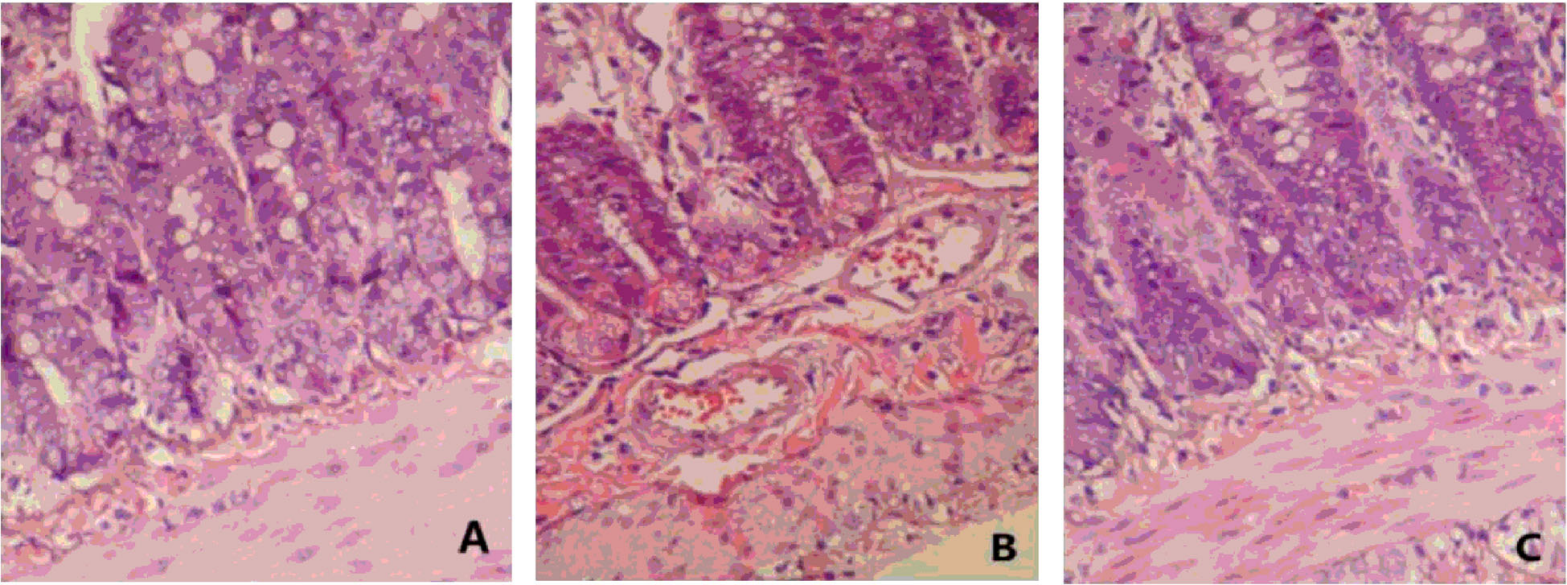
Light micrographs of rat small intestine demonstrating. **A** Normal structure; **B** Enteric tissue after NSAIDs administrating: histology revealed the defect of the villi, epithelial stratification, basal lamina degeneration, and infiltration with neutrophils with diclofenac alone; **C** Enteric tissue after berberine intervention: the above pathological changes were improved than those in the model group

Microscopic examination confirmed that diclofenac could induce severe damage to the rat intestinal mucosa. Histology revealed the villi deficiency, epithelial stratification, basal lamina degeneration, and infiltration with neutrophils. Figure 2 showed an example of lesion in three groups. The results of assessing structural damage were as follows: control group (0.24 ± 0.07), model group (4.41 ± 0.29), berberine group (1.61 ± 0.26). Damage in model group was significantly higher than control group (*P* < 0.05). Compared with model group,the histological scores of berberine were lower (*P* < 0.05) (Fig. 1B). Model rats had obviously shortened and damaged villi in the small intestine.

### The screening of genes and statistical analysis

The results of quantitative gene detection and data analysis was listed in Table 1. We screened HTR4, HTR1A, F2RL3, NOS1, CALCA, NPY, CRHR2, IL1B, P2RX3, NOS2, TPH1, CRHR1, HMOX1, TRPV1, VIP, F2RL1, TGFB1, HTR3A, SLC6A4, TFF2, AQP8 from the small intestine. Rats with NSAID-induced small intestinal disease showed lower levels of NOS1(*P* < 0.05), and higher levels of HTR4, HTR1a, F2RL3, CALCA, NPY, CRHR2, IL1b, P2RX3, TPH1, HMOX1, TRPV1, VIP, F2RL1, SLC6A4, TFF2, AQP8(*P* < 0.05). After berberine intervention, HTR4, F2RL3, NPY, CRHR2, IL1b, VIP, AQP8 were significantly decreased (*P* < 0.05), and NOS1 were significantly increased (*P* < 0.05) (See Table 2).

**Table 1:** Target genes, amplicon sizes, labels, and sequences of primers and probes used in the Luminex assay

Target genes	Amplicon size (bp)	labels	Sequence (5′-3′)
HTR4	132	Forward	TCCTCATGGTGCTGGCCTAT
		Reverse	GATGTGTGCTGTGCTGGTCA
		Probe	AACGGGCAGGAGCCACCTCTGAAAGCA
HTR1a	122	Forward	CTCCACTTTCGGCGCTTTCT
		Reverse	CCGCTCCCTTCTTTTCCACC
		Probe	TCTTCAGAGCCGCGCGCTTCCGAAT
F2RL3	145	Forward	GTACTGTTCTCGGCAGTGGC
		Reverse	CTACGCAGCTGTTGAGGGTG
		Probe	TATGGAGCCTATGTGCCCAGCCTGGCA
NOS1	111	Forward	ATCAGCCAGCAAAGACCAGC
		Reverse	TTAGCCTGGGAGACTGAGCC
		Probe	TCCCAGTAACGGACCTCAGCATGCCCA
CALCA	103	Forward	AACACTGCCACCTGTGTGAC
		Reverse	AGGCTTCAGAGCCCACATTG
		Probe	ATCGGCTGGCAGGTCTGCTGAGCAGAT
NPY	132	Forward	CTGGAGGAGAGCTTGTGGGA
		Reverse	CGCCTACTCCACACTCCTCA
		Probe	ATCGGGCAGGAGCCACCGCTGAAATCA
CRHR2	94	Forward	TTGGATGGTGCATTCCCTGC
		Reverse	CCAGCTTCCTTGCCAAACCA
		Probe	TCATCGCCTGGGCAGTTGGCAAACTCT
IL1b	94	Forward	TCGCAGCAGCACATCAACAA
		Reverse	TCCACGGGAAAGACACAGGT
		Probe	TGGCTGTGGAGAAGCTGTGGCAGCT
P2RX3	93	Forward	GCTGGTATACGGGAACGCTG
		Reverse	AGAACAGTCCCCACTCCCAC
		Probe	ACCATCATCAGCTCGGTGGCTGCCTT
NOS2	80	Forward	ATCACTTCCCCGCCTAGTCC
		Reverse	TCTAGCTCCTGCTGTTCGCT
		Probe	TCGACTGCTCAGCACCCTGGCAGAAGA
TPH1	145	Forward	TGACATCTTCCCCCTGCTGA
		Reverse	CACTCTGTTGGCGCAGAAGT
		Probe	ACTCGCCCGATCAGCTCACTGCGAA
CRHR1	140	Forward	CCTGGCCAGCAATGTCTCTG
		Reverse	TGACGGCAATGTGGTAGTGC
		Probe	ATGGCTACCGGGAATGCCTGGCCAA
HMOX1	137	Forward	TCTGGTATGGGCCTCACTGG
		Reverse	GTCACCCAGGTAGCGGGTAT
		Probe	TCATCCCTTGCACGCCAGCCACACA
TRPV1	137	Forward	TCTGGTATGGGCCTCACTGG
		Reverse	GTCACCCAGGTAGCGGGTAT
		Probe	TCATCCCTTGCACGCCAGCCACACA
VIP	94	Forward	TGGAAGCCAGAAGCAAGCCT
		Reverse	GGTGGTCCAAAGAGAGGCCA
		Probe	AGTGTGCTGTTCTCTCAGTCGCTGGCC
F2RL1	102	Forward	TAATGGCATGGCCCTCTGGA
		Reverse	ATGACAGAGAGGAGGTCGGC
		Probe	ACACCCCGCCGTGATTTACATGGCCA
TGFB1	90	Forward	ATACGCCTGAGTGGCTGTCT
		Reverse	CGCTGAATCGAAAGCCCTGT
		Probe	ACGTCACTGGAGTTGTACGGCAGTGGC
HTR3a	141	Forward	ATAGACCCCCAGCCACCTTC
		Reverse	CCTCCCTTGGTGGTGGAAGA
		Probe	TCAGCCATGGGAAACCACTGCAGCCA
SLC6A4	91	Forward	CATCTTCACGGTGCTTGGCT
		Reverse	GATGAAAAGGAGGCTGGGGC
		Probe	ACGAAGACGTGTCCGAGGTGGCCAAA
TFF2	133	Forward	TCGGAGCAGTGTGTCATGGA
		Reverse	GTGGGAAGAAACACCAGGGC
		Probe	TCGCAAGAATTGTGGGTACCCGGGCA
AQP8	96	Forward	GGCCTTGGGGCTCATCATTG
		Reverse	TTGAGGCCTCCGATCACTGT
		Probe	TCAACCCTGCTGTGTCGCTGGCAGT

**Table 2:** Colonic mucosal gene expression among different groups

	Control group	Model group	Intervention group
HTR4	0.073 ± 0.030	0.383 ± 0.212^a^	0.228 ± 0.074^b^
HTR1a	0.137 ± 0.055	0.608 ± 0.252^a^	0.534 ± 0.353
F2RL3	0.294 ± 0.091	1.523 ± 0.502^a^	0.990 ± 0.295^b^
NOS1	1.254 ± 0.340	0.649 ± 0.289^a^	1.127 ± 0.315^b^
CALCA	0.271 ± 0.123	0.586 ± 0.314^a^	0.578 ± 0.319
NPY	0.243 ± 0.083	1.126 ± 0.354^a^	0.634 ± 0.262^b^
CRHR2	0.201 ± 0.078	0.836 ± 0.333^a^	0.538 ± 0.334^b^
IL1b	0.006 ± 0.001	0.082 ± 0.060^a^	0.044 ± 0.010^b^
P2RX3	0.147 ± 0.059	0.415 ± 0.272^a^	0.366 ± 0.179
NOS2	0.224 ± 0.072	0.517 ± 0.365	0.778 ± 0.443
TPH1	0.033 ± 0.012	0.275 ± 0.231^a^	0.299 ± 0.010
CRHR1	0.005 ± 0.002	0.028 ± 0.033	0.006 ± 0.002
HMOX1	0.495 ± 0.127	1.616 ± 0.624^a^	1.727 ± 0.449
TRPV1	0.053 ± 0.017	0.156 ± 0.087^a^	0.179 ± 0.109
VIP	0.594 ± 0.185	1.110 ± 0.406^a^	0.438 ± 0.167^b^
F2RL1	0.312 ± 0.047	1.451 ± 0.372^a^	1.483 ± 0.460
TGFB1	0.210 ± 0.050	0.205 ± 0.099	0.188 ± 0.050
HTR3a	0.204 ± 0.059	0.162 ± 0.081	0.136 ± 0.062
SLC6a4	0.249 ± 0.086	1.565 ± 0.553^a^	1.416 ± 0.540
TFF2	0.031 ± 0.011	0.114 ± 0.067^a^	0.140 ± 0.088
AQP8	0.227 ± 0.109	0.856 ± 0.253^a^	0.414 ± 0.201^b^

^a^ *P* < 0.05 compared with control group; ^b^ *P* < 0.05 compared with model group

## Discussion

It is revealed that more than 29 million adults (12.1%) regular used NSAIDs and around 43 million adults (19.0%) took aspirin more than 3 months(> 3 times per week). Widespread use of NSAIDs has increased the damage to the digestive tract such as small intestine which worth our attention [3]. Nevertheless, there is no effective treatment for NSAID-induced small intestinal injury because of the complex and indefinite pathogenesis. The aim of this study was to study the specific mechanism of NSAID-induced small intestinal disease and berberine on NSAID-associated small intestinal injury from the gene level. However, previous studies in the literature, including those in our research group, only involved one or two genes. Through literature search, we gathered statistics about differential expression of RNAs in intestinal mucosal tissues and cells in regards to intestinal mucosal barrier injury, intestinal inflammation, gastrointestinal cancer, and inflammatory bowel disease. Therefore, we choose 21 RNAs in rats as study targets [15–26]: HTR4, HTR1a, F2RL3, NOS1, CALCA, NPY, CRHR2, IL1b, P2RX3, NOS2, TPH1, CRHR1, HMOX1, TRPV1, VIP, F2RL1, TGFB1、HTR3a, SLC6A4, TFF2, AQP8. Screening by liquid chip technology, we found that NSAIDs resulted in intestinal damage was associate with HTR4, HTR1a, F2RL3, CALCA, NPY, CRHR2, IL1b, P2RX3, TPH1, HMOX1, TRPV1, VIP, F2RL1, SLC6A4, TFF2, AQP8, NOS1,and berberine treat NSAID- induced small intestinal disease by regulating HTR4, F2RL3, NPY, CRHR2, IL1b, VIP, AQP8, NOS1.

Our experiments have confirmed that the pathogenesis of NSAID-induced small intestinal disease is related to intestinal motility abnormalities, increases permeability of the intestinal mucosa caused by neuroendocrine, inflammatory reactions and disorders of water metabolism. The 5-Hydroxytryptamine Receptor 4(HTR4) and 5-Hydroxytryptamine Receptor 1a(HTR1a) produce a sustained excitatory response by activating protein kinase A and binding to 5-Hydroxytryptamine (5-HT). In this experiment, the expression of HTR4 and HTR1a in the ileum mucosa increased, which suggested that NSAIDs mediated the intestinal nervous system to control the movement of intestinal chromaffin cells and increased the excitability of intestinal motility, and then caused intestinal motility dysfunction [27]. As well as Transient Receptor Potential Cation Channel Subfamily V Member 1(TRPV1) has been found to negatively influence the gastrointestinal muscle contractions [28]. Aquaporin 8(AQP8) proteins was found in the apical brush border membrane of intestinal epithelial cells. Increased AQP8 makes liquid water metabolic abnormalities and disturbs the permeability of intestinal mucosa [29]. Other gene F2RL3, CALCA, NPY, CRHR2, IL1b, P2RX3, TPH1, HMOX1, VIP, F2RL1, SLC6A4, TFF2, NOS1 affect the permeability of intestinal mucosa by different ways and accelerate the occurrence of NSAID-induced intestinal injury [30]. For example, Calcitonin gene-related polypeptide-alpha (afa-CGRP) encoded by the Calcitonin Related Polypeptide Alpha (CALCA) gene is shown a potent vasodilator and can mediate neurogenic inflammation [31].

The liquid chip technology is an easier and more cost-effective alternative to purchasing and running several real-time instruments in parallel. In conclusion, while testing clinical specimens, the Luminex assay shows clinical sensitivity and specificity comparable to real-time Reverse Transcription-Polymerase Chain Reaction (RT-PCR). The assay can process large numbers of specimens at the same time, while significantly reducing the cost. Furthermore, the platform’s flexibility allows for the possibility of adding more targets and of easily changing primer sequences in order to detect more genes at the same time [32].

Berberine, which is extracted from traditional Chinese medicine Rhizoma coptidis, is usually used to treat gastrointestinal disorders, due to its antimicrobial propertie [33]. Recently, berberine is demonstrated to have multiple pharmacological activities, including anticancer effect, regulate metabolism of glucose and lipid. Some study hypothesized that berberine makes its various effects in the intestinal tract before being absorbed [34]. Some studies even found that the structural modified berberine can improve its activity. It is believed that novel anticancer drugs based on the natural product berberine with both anti-inflammation and anti-tumor activities can be developed in the near future [35]. In our experiment, NSAID-induced small intestinal disease model rats intervened by berberine showed significantly change in HTR4, F2RL3, NPY, CRHR2, IL1b, VIP, AQP8, NOS1. Furthermore, in our published studies, we found that berberine could protect the intestinal mucosa of NSAIDs users by up-regulating the expressions of PGP9.5, GFAP and GDNF to repair the enteric nervous system [36]. It is known that HTR4 stimulates cyclic Adenosine monophosphate (cAMP) production in response to serotonin 5-HT. 5-HT plays a role in both the central and peripheral nervous systems, and mediates motility, secretion, vasodilation, as well as sensation in the gastrointestinal tract,and it also protects neuro and promote inflammatory in the gut [37]. Currently, drugs target to 5-HT receptors have been developed to treat the functional gastrointestinal (GI) disorders and pain [38]. 5-HT3 and 5-HT4 receptor subtypes are located in the wall of the gut and have been most extensively studied [37]. The 5-HT4 receptor, is located on enteric nerve terminals to release neurotransmitter when activated, and mediate chloride secretion and goblet cell degranulation, as well as accelerates the peristaltic reflex [39].

Coagulation factor II receptor-like 3 (F2RL3) encodes a member of the protease-activated receptor subfamily, also known as protease-activated receptor 4(PAR4),which takes partin platelet activation, intimal hyperplasia and inflammation [40]. Some study confirmed that PAR4 present in human’s small and large intestine [41]. Recently, it has been found that the neutrophil granule protease cathepsin G3 can stimulate PAR4 [42]. At same time, it links PAR4 activation to the presence of granulocytes [42], which suggests that inflammatory pathophysiological circumstancesmay is associated with PAR4. Previous study showed that IL-1, IL-4, and LPS could increase the expression of Corticotropin Releasing Hormone Receptor 2(CRHR-2) [43]. And CRH secreted after activating CRHR-2 outside the brain had pro-inflammatory actions [44], which could mediate the action of lipopolysaccharide [45], so in intestine CRH-CRHR2 signaling likely played an important role in the progression of inflammation and gastric transit [46]. In human intestine,Interleukin 1 Beta (IL-1b) secretion causes exaggerated inflammatory responses by decreased expression of inhibitors of Nuclear factor kB (NF-kB) [47]. As a known pro-inflammatory cytokines, IL-1B via NF-κB pathway and cytoskeletal contraction and TJ opening to increase intestinal permeability [48].

On the contrary, vasoactive intestinal peptide (VIP) has potent anti-inflammatory effects via downregulates TNF-α expression [49] and NF-ΚB-dependent gene activation [50]. VIP increases expression of the tight junction protein zona and reduces paracellular epithelial permeability in Caco2 and HT29-Cl to indirectly regulate epithelial permeability [50]. Generally speaking, Neuropeptide Y (NPY) and VIP, including their receptors, play a role in inflammatory signaling and epithelial barrier functions.

In humans, AQP8 protein is found as a water selective channel [51] and is located in the absorptive columnar epithelia in duodenum, jejunum, and colon. Many functions of AQPs in the stomach and intestine physiology is confirmed, including water transfer, gastric juice secretion, barrier function [52]. It has been confirmed that AQP8 might be involved in transepithelial water absorption taking place in the small intestine, and the increased expression of AQP8 show to induce small intestine epithelial barrier [53]. Moreover, the anti-diarrhea effects of emodin [54] and berberine [55], may depend on AQPs to regulate water transport and absorption possibly, via PKA/p-CREB signal pathway [54].

## Conclusion

In summary, multiple genes involved in the pathogenesis of NSAID-induced small intestinal disease, associated with intestinal motility disorder, intestinal barrier damage. Chinese medicine berberine has a protective effect on NSAID-associated intestinal injury, the mechanism may be that berberine decreases the expression of intestinal mucosa HTR4, F2RL3, NPY, CRHR2, IL1b, VIP, AQP8, and increases the expression of NOS1 through alleviating inflammation, nerve endocrine regulation to reduce intestinal permeability and protect intestinal mucosal barrier.

## Data Availability

The datasets used and/or analysed during the current study are available from the corresponding author on reasonable request.

## Abbreviations

NSAIDs: Non-steroidal anti-inflammatory drugs
TNBS: 2,4,6-trinitrobenzene sulfonic acid
SD: Sprague Dawley
RNA: Total ribonucleic acid
cDNA: Complementary DNA
HTR4: 5-Hydroxytryptamine Receptor 4
HTR1a: 5-Hydroxytryptamine Receptor 1a
5-HT: 5-Hydroxytryptamine
TRPV1: Transient receptor potential cation channel subfamily V member 1
AQP8: Aquaporin 8
afa-CGRP: Calcitonin gene-related polypeptide-alpha
CALCA: Calcitonin Related Polypeptide Alpha
RT-PCR: Reverse Transcription-Polymerase Chain Reaction
cAMP: Cyclic Adenosine monophosphate
GI: Gastrointestinal
F2RL3: Coagulation factor II receptor-like 3
PAR4: Protease-activated receptor 4
CRHR-2: Corticotropin Releasing Hormone Receptor 2
IL-1b: Interleukin 1 Beta
NF-KB: Nuclear factor kB
VIP: Vasoactive intestinal peptide
NPY: Neuropeptide Y
